# Supplementary material for: Patient and caregiver perceptions of electronic health records interoperability in the NHS and its impact on care quality: a focus group study
Source: BMC Med Inform Decis Mak. 2024 Dec 3;24:370. doi: 10.1186/s12911-024-02789-5 (PMC11616290; doi:10.1186/s12911-024-02789-5)
Supplement: Supplementary file 2 — Supplementary Material 2 [file 12911_2024_2789_MOESM2_ESM.pdf]

Imperial College London

Imperial College Research Ethics Committee
Imperial College London
Room 221
Medical School Building
St Marys Campus
London
W2 1PG
Tel: +44 (0)207 594 1862

researchethicscommittee@imperial.ac.uk

22/02/2022

Dear Dr. Ana Luisa Neves,

Study Title: Patient and caregiver perceptions of electronic health records interoperability and its impact on care quality

ICREC Reference number: 22IC7425

The above study was approved by your Head of Department on 24/12/21 and by the Research Governance and Integrity Team (RGIT) on 22/02/22.

Under the Imperial College Research Ethics Committee process, a study that has been reviewed by the Research Governance and Integrity Team (RGIT) and Head of Division/Department, where no significant ethical issues have been identified in the protocol or ethics application, can be granted RGIT approval without requiring it to go to full committee.

Documents
The documents reviewed were:

- Application form v1.3 21/02/2022
- Protocol v1 10/12/2021
- Participant Information Sheet v1.2 14/02/2022
- Consent form V1 13/12/2021
- Invitation E-mail v1 22/02/2022
- Topic Guide v1 14/02/2022

Yours sincerely

Ruth Nicholson Digitally signed by Ruth Nicholson
Date: 2022.02.22 15:29:46 Z

Ruth Nicholson
Head of Research Governance and Integrity
Imperial College London
